# Supplementary material for: Modeling glioblastoma heterogeneity as a dynamic network of cell states
Source: Mol Syst Biol. 2021 Sep 16;17(9):e10105. doi: 10.15252/msb.202010105 (PMC8444284; doi:10.15252/msb.202010105)
Supplement: Supplementary file 5 — Source Data for Figure 3 [file MSB-17-e10105-s001.zip › Figure3A_sourcedata/GSEA_3065/nosigdb_state1.GseaPreranked.1622718409535/DOROSZKO_U3180MG_CLUSTER_3.html]

Details for gene set DOROSZKO\_U3180MG\_CLUSTER\_3[GSEA]

|  || Dataset | state1 |
| Phenotype | NoPhenotypeAvailable |
| Upregulated in class | na\_pos |
| GeneSet | DOROSZKO\_U3180MG\_CLUSTER\_3 |
| Enrichment Score (ES) | 0.5000404 |
| Normalized Enrichment Score (NES) | 2.1413417 |
| Nominal p-value | 0.0 |
| FDR q-value | 0.0 |
| FWER p-Value | 0.0 |
Table: GSEA Results Summary

  

Fig 1: Enrichment plot: DOROSZKO\_U3180MG\_CLUSTER\_3      
 Profile of the Running ES Score & Positions of GeneSet Members on the Rank Ordered List

  

| PROBE | GENE SYMBOL | GENE\_TITLE | RANK IN GENE LIST | RANK METRIC SCORE | RUNNING ES | CORE ENRICHMENT || 1 | HMGA1 |  |  | 8 | 0.736 | 0.0147 | Yes |
| 2 | SMS |  |  | 23 | 0.619 | 0.0262 | Yes |
| 3 | TUBA1C |  |  | 24 | 0.619 | 0.0393 | Yes |
| 4 | CSRP2 |  |  | 28 | 0.574 | 0.0511 | Yes |
| 5 | PLP2 |  |  | 59 | 0.446 | 0.0573 | Yes |
| 6 | TUBB4B |  |  | 67 | 0.429 | 0.0656 | Yes |
| 7 | CLIC1 |  |  | 76 | 0.406 | 0.0733 | Yes |
| 8 | ARPC1B |  |  | 108 | 0.358 | 0.0775 | Yes |
| 9 | PDCD5 |  |  | 109 | 0.358 | 0.0851 | Yes |
| 10 | COX5A |  |  | 116 | 0.348 | 0.0918 | Yes |
| 11 | CTNNAL1 |  |  | 123 | 0.343 | 0.0984 | Yes |
| 12 | UBE2C |  |  | 125 | 0.340 | 0.1054 | Yes |
| 13 | ITGB1BP1 |  |  | 126 | 0.339 | 0.1126 | Yes |
| 14 | H2AFZ |  |  | 153 | 0.321 | 0.1166 | Yes |
| 15 | RANBP1 |  |  | 154 | 0.320 | 0.1233 | Yes |
| 16 | LSM4 |  |  | 162 | 0.316 | 0.1292 | Yes |
| 17 | SNRPB |  |  | 172 | 0.310 | 0.1348 | Yes |
| 18 | CDKN3 |  |  | 178 | 0.307 | 0.1407 | Yes |
| 19 | PGAM1 |  |  | 190 | 0.302 | 0.1459 | Yes |
| 20 | CKS2 |  |  | 195 | 0.297 | 0.1517 | Yes |
| 21 | EEF1B2 |  |  | 197 | 0.296 | 0.1579 | Yes |
| 22 | BIRC5 |  |  | 202 | 0.295 | 0.1637 | Yes |
| 23 | NRGN |  |  | 204 | 0.294 | 0.1698 | Yes |
| 24 | DTYMK |  |  | 205 | 0.292 | 0.1759 | Yes |
| 25 | EIF4EBP1 |  |  | 216 | 0.290 | 0.1810 | Yes |
| 26 | MRPL17 |  |  | 226 | 0.285 | 0.1860 | Yes |
| 27 | MZT2B |  |  | 232 | 0.283 | 0.1914 | Yes |
| 28 | POP7 |  |  | 236 | 0.282 | 0.1971 | Yes |
| 29 | MEA1 |  |  | 243 | 0.279 | 0.2023 | Yes |
| 30 | MINOS1 |  |  | 250 | 0.277 | 0.2075 | Yes |
| 31 | AP2S1 |  |  | 251 | 0.276 | 0.2133 | Yes |
| 32 | SIVA1 |  |  | 252 | 0.276 | 0.2192 | Yes |
| 33 | PIN1 |  |  | 264 | 0.274 | 0.2238 | Yes |
| 34 | DEK |  |  | 296 | 0.261 | 0.2260 | Yes |
| 35 | GTF3A |  |  | 310 | 0.257 | 0.2300 | Yes |
| 36 | CKS1B |  |  | 346 | 0.242 | 0.2314 | Yes |
| 37 | NDUFA6 |  |  | 347 | 0.242 | 0.2365 | Yes |
| 38 | LSM3 |  |  | 361 | 0.237 | 0.2401 | Yes |
| 39 | SNRNP25 |  |  | 367 | 0.236 | 0.2445 | Yes |
| 40 | ALYREF |  |  | 371 | 0.234 | 0.2491 | Yes |
| 41 | BANF1 |  |  | 373 | 0.233 | 0.2539 | Yes |
| 42 | PPP1CA |  |  | 377 | 0.232 | 0.2585 | Yes |
| 43 | ACAT2 |  |  | 385 | 0.229 | 0.2626 | Yes |
| 44 | NME1 |  |  | 395 | 0.227 | 0.2664 | Yes |
| 45 | TUBB |  |  | 398 | 0.226 | 0.2710 | Yes |
| 46 | CHCHD3 |  |  | 402 | 0.226 | 0.2754 | Yes |
| 47 | ARPC5L |  |  | 409 | 0.224 | 0.2795 | Yes |
| 48 | PHF19 |  |  | 415 | 0.223 | 0.2837 | Yes |
| 49 | HNRNPAB |  |  | 430 | 0.221 | 0.2869 | Yes |
| 50 | TUBG1 |  |  | 439 | 0.219 | 0.2906 | Yes |
| 51 | CCT5 |  |  | 440 | 0.218 | 0.2952 | Yes |
| 52 | CACYBP |  |  | 458 | 0.214 | 0.2979 | Yes |
| 53 | MRPL18 |  |  | 470 | 0.212 | 0.3012 | Yes |
| 54 | TMEM106C |  |  | 478 | 0.211 | 0.3049 | Yes |
| 55 | BCL7C |  |  | 489 | 0.208 | 0.3082 | Yes |
| 56 | PSMA3 |  |  | 528 | 0.200 | 0.3084 | Yes |
| 57 | H2AFV |  |  | 538 | 0.198 | 0.3116 | Yes |
| 58 | TPX2 |  |  | 556 | 0.194 | 0.3139 | Yes |
| 59 | MZT2A |  |  | 564 | 0.191 | 0.3172 | Yes |
| 60 | DCTPP1 |  |  | 566 | 0.191 | 0.3211 | Yes |
| 61 | NFIC |  |  | 568 | 0.190 | 0.3250 | Yes |
| 62 | MRPL37 |  |  | 589 | 0.188 | 0.3268 | Yes |
| 63 | PFKP |  |  | 594 | 0.187 | 0.3303 | Yes |
| 64 | MAD2L2 |  |  | 607 | 0.185 | 0.3330 | Yes |
| 65 | SNRPD3 |  |  | 616 | 0.183 | 0.3360 | Yes |
| 66 | YWHAQ |  |  | 618 | 0.183 | 0.3397 | Yes |
| 67 | ANP32B |  |  | 622 | 0.183 | 0.3432 | Yes |
| 68 | OLA1 |  |  | 630 | 0.181 | 0.3463 | Yes |
| 69 | SNRPD1 |  |  | 647 | 0.179 | 0.3484 | Yes |
| 70 | LY6E |  |  | 649 | 0.179 | 0.3521 | Yes |
| 71 | CENPX |  |  | 669 | 0.176 | 0.3538 | Yes |
| 72 | SNRPF |  |  | 673 | 0.176 | 0.3571 | Yes |
| 73 | PSMC3 |  |  | 692 | 0.173 | 0.3589 | Yes |
| 74 | VOPP1 |  |  | 719 | 0.170 | 0.3597 | Yes |
| 75 | CMC2 |  |  | 722 | 0.169 | 0.3630 | Yes |
| 76 | TMEM14C |  |  | 730 | 0.167 | 0.3658 | Yes |
| 77 | CSNK2B |  |  | 733 | 0.167 | 0.3691 | Yes |
| 78 | TMEM237 |  |  | 735 | 0.167 | 0.3725 | Yes |
| 79 | PSMB9 |  |  | 745 | 0.166 | 0.3751 | Yes |
| 80 | MCUR1 |  |  | 758 | 0.165 | 0.3773 | Yes |
| 81 | CENPN |  |  | 764 | 0.163 | 0.3802 | Yes |
| 82 | PA2G4 |  |  | 780 | 0.160 | 0.3819 | Yes |
| 83 | NABP2 |  |  | 785 | 0.160 | 0.3849 | Yes |
| 84 | KRBOX1 |  |  | 788 | 0.158 | 0.3880 | Yes |
| 85 | VPS29 |  |  | 817 | 0.155 | 0.3883 | Yes |
| 86 | ATP5MC1 |  |  | 820 | 0.154 | 0.3913 | Yes |
| 87 | NUDC |  |  | 835 | 0.153 | 0.3931 | Yes |
| 88 | GAMT |  |  | 843 | 0.152 | 0.3955 | Yes |
| 89 | GLO1 |  |  | 855 | 0.151 | 0.3975 | Yes |
| 90 | EEF1E1 |  |  | 857 | 0.150 | 0.4006 | Yes |
| 91 | CCT4 |  |  | 858 | 0.150 | 0.4038 | Yes |
| 92 | CDCA2 |  |  | 859 | 0.150 | 0.4069 | Yes |
| 93 | SAE1 |  |  | 863 | 0.150 | 0.4098 | Yes |
| 94 | PSMG1 |  |  | 871 | 0.148 | 0.4121 | Yes |
| 95 | PGK1 |  |  | 875 | 0.148 | 0.4149 | Yes |
| 96 | POLR2I |  |  | 878 | 0.147 | 0.4178 | Yes |
| 97 | HMGB3 |  |  | 907 | 0.144 | 0.4179 | Yes |
| 98 | POLE4 |  |  | 910 | 0.143 | 0.4207 | Yes |
| 99 | MCUB |  |  | 916 | 0.143 | 0.4232 | Yes |
| 100 | TUBB6 |  |  | 931 | 0.142 | 0.4247 | Yes |
| 101 | UQCC2 |  |  | 934 | 0.141 | 0.4274 | Yes |
| 102 | SVIP |  |  | 937 | 0.141 | 0.4302 | Yes |
| 103 | CBR3 |  |  | 954 | 0.139 | 0.4314 | Yes |
| 104 | RALY |  |  | 961 | 0.138 | 0.4337 | Yes |
| 105 | WDR34 |  |  | 972 | 0.137 | 0.4355 | Yes |
| 106 | GGCT |  |  | 974 | 0.137 | 0.4383 | Yes |
| 107 | SAC3D1 |  |  | 976 | 0.137 | 0.4411 | Yes |
| 108 | LMNB2 |  |  | 991 | 0.134 | 0.4424 | Yes |
| 109 | NME4 |  |  | 992 | 0.134 | 0.4453 | Yes |
| 110 | CDCA3 |  |  | 994 | 0.134 | 0.4480 | Yes |
| 111 | SLC20A1 |  |  | 1019 | 0.132 | 0.4482 | Yes |
| 112 | XRCC6 |  |  | 1025 | 0.131 | 0.4504 | Yes |
| 113 | H2AFY |  |  | 1037 | 0.130 | 0.4520 | Yes |
| 114 | RPA3 |  |  | 1039 | 0.129 | 0.4546 | Yes |
| 115 | MAGOHB |  |  | 1047 | 0.128 | 0.4566 | Yes |
| 116 | SSRP1 |  |  | 1051 | 0.128 | 0.4590 | Yes |
| 117 | TK1 |  |  | 1056 | 0.127 | 0.4612 | Yes |
| 118 | COPS3 |  |  | 1059 | 0.127 | 0.4637 | Yes |
| 119 | AKR7A2 |  |  | 1091 | 0.124 | 0.4630 | Yes |
| 120 | RFC2 |  |  | 1094 | 0.123 | 0.4654 | Yes |
| 121 | TALDO1 |  |  | 1097 | 0.123 | 0.4677 | Yes |
| 122 | MRPS26 |  |  | 1109 | 0.121 | 0.4691 | Yes |
| 123 | NUDT1 |  |  | 1144 | 0.118 | 0.4680 | Yes |
| 124 | AK2 |  |  | 1167 | 0.116 | 0.4681 | Yes |
| 125 | SUMO3 |  |  | 1190 | 0.113 | 0.4682 | Yes |
| 126 | TPRKB |  |  | 1211 | 0.111 | 0.4684 | Yes |
| 127 | SLBP |  |  | 1219 | 0.111 | 0.4700 | Yes |
| 128 | CMSS1 |  |  | 1229 | 0.110 | 0.4713 | Yes |
| 129 | CENPU |  |  | 1249 | 0.108 | 0.4716 | Yes |
| 130 | DDX39A |  |  | 1255 | 0.108 | 0.4733 | Yes |
| 131 | CENPW |  |  | 1278 | 0.106 | 0.4732 | Yes |
| 132 | KIF4A |  |  | 1303 | 0.103 | 0.4728 | Yes |
| 133 | C3orf14 |  |  | 1309 | 0.103 | 0.4745 | Yes |
| 134 | TFDP1 |  |  | 1318 | 0.102 | 0.4758 | Yes |
| 135 | HSPB11 |  |  | 1325 | 0.101 | 0.4773 | Yes |
| 136 | ACTL6A |  |  | 1333 | 0.100 | 0.4786 | Yes |
| 137 | GPN3 |  |  | 1354 | 0.099 | 0.4786 | Yes |
| 138 | CKAP2L |  |  | 1359 | 0.099 | 0.4802 | Yes |
| 139 | CENPV |  |  | 1361 | 0.098 | 0.4822 | Yes |
| 140 | FKBP3 |  |  | 1366 | 0.098 | 0.4839 | Yes |
| 141 | UBA2 |  |  | 1380 | 0.097 | 0.4845 | Yes |
| 142 | CCND3 |  |  | 1389 | 0.096 | 0.4857 | Yes |
| 143 | MAD2L1 |  |  | 1394 | 0.095 | 0.4873 | Yes |
| 144 | DHFR |  |  | 1413 | 0.094 | 0.4873 | Yes |
| 145 | UBE2T |  |  | 1440 | 0.093 | 0.4865 | Yes |
| 146 | ZWILCH |  |  | 1449 | 0.092 | 0.4876 | Yes |
| 147 | PPIH |  |  | 1456 | 0.092 | 0.4889 | Yes |
| 148 | DEPDC1 |  |  | 1478 | 0.090 | 0.4886 | Yes |
| 149 | H2AFX |  |  | 1479 | 0.090 | 0.4905 | Yes |
| 150 | TIMM10 |  |  | 1484 | 0.090 | 0.4920 | Yes |
| 151 | TYMS |  |  | 1501 | 0.089 | 0.4921 | Yes |
| 152 | HNRNPD |  |  | 1509 | 0.088 | 0.4933 | Yes |
| 153 | TEX30 |  |  | 1547 | 0.086 | 0.4911 | Yes |
| 154 | TCTEX1D2 |  |  | 1566 | 0.085 | 0.4910 | Yes |
| 155 | HMGB2 |  |  | 1570 | 0.085 | 0.4925 | Yes |
| 156 | PXMP2 |  |  | 1573 | 0.085 | 0.4941 | Yes |
| 157 | DNAJC9 |  |  | 1594 | 0.083 | 0.4937 | Yes |
| 158 | HIST1H1E |  |  | 1608 | 0.082 | 0.4940 | Yes |
| 159 | KIF23 |  |  | 1638 | 0.080 | 0.4926 | Yes |
| 160 | PBK |  |  | 1651 | 0.079 | 0.4930 | Yes |
| 161 | POLE3 |  |  | 1657 | 0.078 | 0.4941 | Yes |
| 162 | ECI1 |  |  | 1698 | 0.076 | 0.4915 | Yes |
| 163 | ANP32E |  |  | 1706 | 0.075 | 0.4923 | Yes |
| 164 | EIF4A3 |  |  | 1725 | 0.074 | 0.4919 | Yes |
| 165 | NUSAP1 |  |  | 1736 | 0.073 | 0.4924 | Yes |
| 166 | PSMG2 |  |  | 1743 | 0.073 | 0.4933 | Yes |
| 167 | H1FX |  |  | 1752 | 0.072 | 0.4940 | Yes |
| 168 | PIMREG |  |  | 1777 | 0.071 | 0.4929 | Yes |
| 169 | PRC1 |  |  | 1794 | 0.070 | 0.4927 | Yes |
| 170 | NDUFB11 |  |  | 1823 | 0.068 | 0.4912 | Yes |
| 171 | PAFAH1B3 |  |  | 1834 | 0.068 | 0.4915 | Yes |
| 172 | CCNA2 |  |  | 1835 | 0.068 | 0.4930 | Yes |
| 173 | CKLF |  |  | 1837 | 0.068 | 0.4943 | Yes |
| 174 | EXOSC8 |  |  | 1851 | 0.067 | 0.4943 | Yes |
| 175 | KIF22 |  |  | 1858 | 0.067 | 0.4951 | Yes |
| 176 | TCF19 |  |  | 1866 | 0.067 | 0.4957 | Yes |
| 177 | RAD51C |  |  | 1874 | 0.066 | 0.4964 | Yes |
| 178 | SF3B4 |  |  | 1884 | 0.066 | 0.4968 | Yes |
| 179 | SRPK1 |  |  | 1900 | 0.065 | 0.4966 | Yes |
| 180 | USP1 |  |  | 1921 | 0.064 | 0.4958 | Yes |
| 181 | ZWINT |  |  | 1923 | 0.064 | 0.4971 | Yes |
| 182 | TACC3 |  |  | 1949 | 0.063 | 0.4957 | Yes |
| 183 | PRADC1 |  |  | 1980 | 0.060 | 0.4938 | Yes |
| 184 | HNRNPA3 |  |  | 1989 | 0.060 | 0.4942 | Yes |
| 185 | RACGAP1 |  |  | 1994 | 0.060 | 0.4951 | Yes |
| 186 | CDK4 |  |  | 2000 | 0.060 | 0.4958 | Yes |
| 187 | COMMD4 |  |  | 2009 | 0.059 | 0.4962 | Yes |
| 188 | TRAP1 |  |  | 2011 | 0.059 | 0.4973 | Yes |
| 189 | TMUB1 |  |  | 2012 | 0.059 | 0.4986 | Yes |
| 190 | JPT2 |  |  | 2015 | 0.059 | 0.4996 | Yes |
| 191 | PSMD14 |  |  | 2025 | 0.058 | 0.4999 | Yes |
| 192 | NUDT5 |  |  | 2036 | 0.058 | 0.5000 | Yes |
| 193 | CDCA4 |  |  | 2073 | 0.056 | 0.4974 | No |
| 194 | VKORC1 |  |  | 2076 | 0.056 | 0.4984 | No |
| 195 | RCC1 |  |  | 2082 | 0.056 | 0.4990 | No |
| 196 | MIS18A |  |  | 2094 | 0.055 | 0.4990 | No |
| 197 | GMNN |  |  | 2097 | 0.055 | 0.4999 | No |
| 198 | BAZ1B |  |  | 2108 | 0.055 | 0.5000 | No |
| 199 | KPNB1 |  |  | 2179 | 0.052 | 0.4937 | No |
| 200 | RBBP8 |  |  | 2212 | 0.051 | 0.4913 | No |
| 201 | CENPH |  |  | 2215 | 0.051 | 0.4922 | No |
| 202 | KIF2C |  |  | 2224 | 0.050 | 0.4924 | No |
| 203 | PMVK |  |  | 2246 | 0.049 | 0.4912 | No |
| 204 | GNG4 |  |  | 2285 | 0.048 | 0.4882 | No |
| 205 | UBR7 |  |  | 2306 | 0.047 | 0.4870 | No |
| 206 | SMC4 |  |  | 2308 | 0.047 | 0.4879 | No |
| 207 | TRIP13 |  |  | 2314 | 0.047 | 0.4884 | No |
| 208 | ACYP1 |  |  | 2324 | 0.046 | 0.4884 | No |
| 209 | CENPM |  |  | 2348 | 0.045 | 0.4869 | No |
| 210 | DPM2 |  |  | 2357 | 0.045 | 0.4870 | No |
| 211 | RPA2 |  |  | 2385 | 0.044 | 0.4850 | No |
| 212 | SUZ12 |  |  | 2408 | 0.043 | 0.4836 | No |
| 213 | FEN1 |  |  | 2420 | 0.043 | 0.4833 | No |
| 214 | POC1A |  |  | 2424 | 0.042 | 0.4839 | No |
| 215 | PPIF |  |  | 2457 | 0.041 | 0.4814 | No |
| 216 | AHCY |  |  | 2474 | 0.041 | 0.4805 | No |
| 217 | BTG3 |  |  | 2514 | 0.039 | 0.4772 | No |
| 218 | LRR1 |  |  | 2521 | 0.039 | 0.4774 | No |
| 219 | MRPS11 |  |  | 2533 | 0.038 | 0.4770 | No |
| 220 | HNRNPF |  |  | 2541 | 0.038 | 0.4771 | No |
| 221 | MYBL2 |  |  | 2549 | 0.038 | 0.4772 | No |
| 222 | GINS2 |  |  | 2567 | 0.038 | 0.4761 | No |
| 223 | DNMT1 |  |  | 2576 | 0.037 | 0.4761 | No |
| 224 | CTPS1 |  |  | 2594 | 0.036 | 0.4750 | No |
| 225 | ETFB |  |  | 2629 | 0.035 | 0.4722 | No |
| 226 | NCAPH2 |  |  | 2656 | 0.034 | 0.4701 | No |
| 227 | MCMBP |  |  | 2663 | 0.034 | 0.4702 | No |
| 228 | YEATS4 |  |  | 2690 | 0.033 | 0.4681 | No |
| 229 | GTSE1 |  |  | 2703 | 0.033 | 0.4676 | No |
| 230 | NCAPG2 |  |  | 2704 | 0.033 | 0.4682 | No |
| 231 | IER3IP1 |  |  | 2719 | 0.032 | 0.4674 | No |
| 232 | HDAC2 |  |  | 2724 | 0.032 | 0.4677 | No |
| 233 | UNG |  |  | 2727 | 0.032 | 0.4682 | No |
| 234 | DBF4 |  |  | 2751 | 0.031 | 0.4664 | No |
| 235 | MRM2 |  |  | 2759 | 0.031 | 0.4663 | No |
| 236 | MKI67 |  |  | 2760 | 0.031 | 0.4669 | No |
| 237 | CKAP2 |  |  | 2763 | 0.031 | 0.4674 | No |
| 238 | PSMC3IP |  |  | 2766 | 0.031 | 0.4678 | No |
| 239 | ARHGAP11A |  |  | 2814 | 0.030 | 0.4635 | No |
| 240 | CARHSP1 |  |  | 2827 | 0.030 | 0.4628 | No |
| 241 | RAD1 |  |  | 2856 | 0.029 | 0.4605 | No |
| 242 | FBXO5 |  |  | 2870 | 0.028 | 0.4597 | No |
| 243 | LSM8 |  |  | 2892 | 0.028 | 0.4580 | No |
| 244 | ASF1B |  |  | 2929 | 0.027 | 0.4548 | No |
| 245 | MAPRE1 |  |  | 2941 | 0.026 | 0.4541 | No |
| 246 | INAFM1 |  |  | 2954 | 0.026 | 0.4534 | No |
| 247 | NUDT15 |  |  | 2985 | 0.025 | 0.4508 | No |
| 248 | DUT |  |  | 3005 | 0.025 | 0.4493 | No |
| 249 | CIP2A |  |  | 3010 | 0.025 | 0.4494 | No |
| 250 | NUDT21 |  |  | 3013 | 0.025 | 0.4497 | No |
| 251 | RBBP7 |  |  | 3019 | 0.024 | 0.4496 | No |
| 252 | DERA |  |  | 3045 | 0.024 | 0.4475 | No |
| 253 | TMPO |  |  | 3093 | 0.023 | 0.4430 | No |
| 254 | FOXM1 |  |  | 3117 | 0.022 | 0.4410 | No |
| 255 | DCK |  |  | 3176 | 0.021 | 0.4353 | No |
| 256 | NR2C2AP |  |  | 3190 | 0.021 | 0.4343 | No |
| 257 | HAUS1 |  |  | 3219 | 0.020 | 0.4318 | No |
| 258 | RPA1 |  |  | 3239 | 0.020 | 0.4302 | No |
| 259 | VRK1 |  |  | 3288 | 0.018 | 0.4254 | No |
| 260 | SKA1 |  |  | 3345 | 0.017 | 0.4198 | No |
| 261 | OIP5 |  |  | 3357 | 0.017 | 0.4190 | No |
| 262 | PARPBP |  |  | 3380 | 0.016 | 0.4170 | No |
| 263 | SKA3 |  |  | 3384 | 0.016 | 0.4171 | No |
| 264 | CDK1 |  |  | 3396 | 0.016 | 0.4162 | No |
| 265 | SPC24 |  |  | 3404 | 0.016 | 0.4158 | No |
| 266 | PCLAF |  |  | 3474 | 0.014 | 0.4088 | No |
| 267 | PCNA |  |  | 3540 | 0.013 | 0.4022 | No |
| 268 | CHAF1B |  |  | 3551 | 0.013 | 0.4014 | No |
| 269 | FANCG |  |  | 3562 | 0.013 | 0.4006 | No |
| 270 | HIST1H1D |  |  | 3604 | 0.012 | 0.3964 | No |
| 271 | CHAC2 |  |  | 3626 | 0.011 | 0.3945 | No |
| 272 | SHCBP1 |  |  | 3637 | 0.011 | 0.3936 | No |
| 273 | RFC5 |  |  | 3697 | 0.010 | 0.3876 | No |
| 274 | FARSB |  |  | 3754 | 0.009 | 0.3818 | No |
| 275 | HJURP |  |  | 3796 | 0.008 | 0.3776 | No |
| 276 | RAB23 |  |  | 3816 | 0.008 | 0.3758 | No |
| 277 | SHMT2 |  |  | 3865 | 0.007 | 0.3708 | No |
| 278 | CCDC167 |  |  | 3921 | 0.006 | 0.3651 | No |
| 279 | NRM |  |  | 3968 | 0.005 | 0.3603 | No |
| 280 | NCAPH |  |  | 4028 | 0.004 | 0.3541 | No |
| 281 | COA1 |  |  | 4046 | 0.003 | 0.3524 | No |
| 282 | DEPDC1B |  |  | 4058 | 0.003 | 0.3512 | No |
| 283 | DONSON |  |  | 4072 | 0.003 | 0.3499 | No |
| 284 | TMEM54 |  |  | 4075 | 0.003 | 0.3498 | No |
| 285 | UHRF1 |  |  | 4092 | 0.003 | 0.3481 | No |
| 286 | ARRB2 |  |  | 4160 | 0.001 | 0.3410 | No |
| 287 | HIRIP3 |  |  | 4163 | 0.001 | 0.3409 | No |
| 288 | SGO2 |  |  | 4184 | 0.001 | 0.3388 | No |
| 289 | CDCA5 |  |  | 4222 | 0.000 | 0.3348 | No |
| 290 | NCAPG |  |  | 4261 | -0.000 | 0.3308 | No |
| 291 | SUPT16H |  |  | 4275 | -0.001 | 0.3294 | No |
| 292 | HAUS2 |  |  | 4309 | -0.001 | 0.3259 | No |
| 293 | NUF2 |  |  | 4342 | -0.002 | 0.3226 | No |
| 294 | PRTFDC1 |  |  | 4344 | -0.002 | 0.3225 | No |
| 295 | DSCC1 |  |  | 4360 | -0.002 | 0.3210 | No |
| 296 | FDPS |  |  | 4450 | -0.004 | 0.3116 | No |
| 297 | P4HB |  |  | 4459 | -0.004 | 0.3108 | No |
| 298 | PAXX |  |  | 4467 | -0.004 | 0.3101 | No |
| 299 | RNASEH2B |  |  | 4493 | -0.004 | 0.3076 | No |
| 300 | POLA2 |  |  | 4497 | -0.004 | 0.3073 | No |
| 301 | C5orf34 |  |  | 4502 | -0.005 | 0.3070 | No |
| 302 | NFYB |  |  | 4506 | -0.005 | 0.3068 | No |
| 303 | CNPY3 |  |  | 4517 | -0.005 | 0.3058 | No |
| 304 | SAAL1 |  |  | 4532 | -0.005 | 0.3044 | No |
| 305 | CD151 |  |  | 4546 | -0.005 | 0.3032 | No |
| 306 | MELK |  |  | 4558 | -0.006 | 0.3021 | No |
| 307 | FAF1 |  |  | 4573 | -0.006 | 0.3008 | No |
| 308 | MGME1 |  |  | 4577 | -0.006 | 0.3006 | No |
| 309 | HPF1 |  |  | 4607 | -0.006 | 0.2976 | No |
| 310 | MCM3 |  |  | 4633 | -0.007 | 0.2951 | No |
| 311 | WDHD1 |  |  | 4656 | -0.007 | 0.2929 | No |
| 312 | CENPK |  |  | 4697 | -0.007 | 0.2888 | No |
| 313 | SPC25 |  |  | 4740 | -0.008 | 0.2845 | No |
| 314 | AURKB |  |  | 4764 | -0.008 | 0.2822 | No |
| 315 | BUB3 |  |  | 4828 | -0.010 | 0.2757 | No |
| 316 | IMMT |  |  | 4844 | -0.010 | 0.2744 | No |
| 317 | RAD21 |  |  | 4916 | -0.011 | 0.2671 | No |
| 318 | LRRC45 |  |  | 4928 | -0.011 | 0.2661 | No |
| 319 | CHAF1A |  |  | 4943 | -0.012 | 0.2649 | No |
| 320 | MND1 |  |  | 4982 | -0.012 | 0.2611 | No |
| 321 | XRN2 |  |  | 5015 | -0.013 | 0.2580 | No |
| 322 | KIFC1 |  |  | 5017 | -0.013 | 0.2581 | No |
| 323 | TEDC1 |  |  | 5046 | -0.013 | 0.2554 | No |
| 324 | TIMELESS |  |  | 5058 | -0.013 | 0.2546 | No |
| 325 | CCNE2 |  |  | 5070 | -0.014 | 0.2537 | No |
| 326 | RAC3 |  |  | 5075 | -0.014 | 0.2535 | No |
| 327 | PANK2 |  |  | 5090 | -0.014 | 0.2523 | No |
| 328 | MXD3 |  |  | 5096 | -0.014 | 0.2521 | No |
| 329 | SCCPDH |  |  | 5110 | -0.014 | 0.2510 | No |
| 330 | BUB1B |  |  | 5187 | -0.015 | 0.2433 | No |
| 331 | PHGDH |  |  | 5207 | -0.016 | 0.2416 | No |
| 332 | TEDC2 |  |  | 5221 | -0.016 | 0.2405 | No |
| 333 | RAD18 |  |  | 5244 | -0.016 | 0.2385 | No |
| 334 | DSN1 |  |  | 5258 | -0.016 | 0.2375 | No |
| 335 | CCDC34 |  |  | 5298 | -0.017 | 0.2337 | No |
| 336 | CHEK1 |  |  | 5330 | -0.017 | 0.2307 | No |
| 337 | CISD2 |  |  | 5358 | -0.018 | 0.2282 | No |
| 338 | SRR |  |  | 5410 | -0.019 | 0.2232 | No |
| 339 | TECR |  |  | 5413 | -0.019 | 0.2234 | No |
| 340 | PAQR4 |  |  | 5415 | -0.019 | 0.2237 | No |
| 341 | MYBL1 |  |  | 5455 | -0.019 | 0.2199 | No |
| 342 | KPNA2 |  |  | 5461 | -0.020 | 0.2198 | No |
| 343 | CCT2 |  |  | 5479 | -0.020 | 0.2184 | No |
| 344 | RMI2 |  |  | 5517 | -0.020 | 0.2149 | No |
| 345 | HAUS8 |  |  | 5537 | -0.021 | 0.2133 | No |
| 346 | ASRGL1 |  |  | 5584 | -0.022 | 0.2089 | No |
| 347 | MCM5 |  |  | 5588 | -0.022 | 0.2090 | No |
| 348 | MTHFD1 |  |  | 5596 | -0.022 | 0.2088 | No |
| 349 | CDT1 |  |  | 5644 | -0.023 | 0.2042 | No |
| 350 | ITGB3BP |  |  | 5717 | -0.024 | 0.1971 | No |
| 351 | RECQL4 |  |  | 5723 | -0.024 | 0.1971 | No |
| 352 | PSIP1 |  |  | 5794 | -0.025 | 0.1902 | No |
| 353 | WDR54 |  |  | 5844 | -0.026 | 0.1855 | No |
| 354 | CLSPN |  |  | 5848 | -0.026 | 0.1857 | No |
| 355 | RTKN2 |  |  | 5868 | -0.027 | 0.1843 | No |
| 356 | PKMYT1 |  |  | 5879 | -0.027 | 0.1838 | No |
| 357 | PIDD1 |  |  | 5961 | -0.029 | 0.1758 | No |
| 358 | CDK2 |  |  | 5964 | -0.029 | 0.1762 | No |
| 359 | ACADM |  |  | 5968 | -0.029 | 0.1764 | No |
| 360 | CEP57 |  |  | 5983 | -0.029 | 0.1756 | No |
| 361 | PARP2 |  |  | 5986 | -0.029 | 0.1760 | No |
| 362 | RFC3 |  |  | 6030 | -0.030 | 0.1720 | No |
| 363 | DIAPH3 |  |  | 6103 | -0.031 | 0.1650 | No |
| 364 | CHTF18 |  |  | 6113 | -0.031 | 0.1647 | No |
| 365 | PRIM2 |  |  | 6118 | -0.031 | 0.1649 | No |
| 366 | GINS1 |  |  | 6149 | -0.032 | 0.1624 | No |
| 367 | RRM2 |  |  | 6214 | -0.033 | 0.1563 | No |
| 368 | NDC80 |  |  | 6240 | -0.034 | 0.1544 | No |
| 369 | E2F1 |  |  | 6247 | -0.034 | 0.1544 | No |
| 370 | SCARA3 |  |  | 6250 | -0.034 | 0.1549 | No |
| 371 | CENPQ |  |  | 6262 | -0.034 | 0.1545 | No |
| 372 | MCM2 |  |  | 6282 | -0.034 | 0.1532 | No |
| 373 | WDR76 |  |  | 6347 | -0.036 | 0.1471 | No |
| 374 | DTL |  |  | 6465 | -0.038 | 0.1355 | No |
| 375 | MACROD1 |  |  | 6469 | -0.038 | 0.1360 | No |
| 376 | KNL1 |  |  | 6527 | -0.039 | 0.1308 | No |
| 377 | ENOPH1 |  |  | 6568 | -0.040 | 0.1273 | No |
| 378 | MSH6 |  |  | 6591 | -0.040 | 0.1259 | No |
| 379 | FBXO4 |  |  | 6658 | -0.042 | 0.1197 | No |
| 380 | XRCC5 |  |  | 6701 | -0.043 | 0.1162 | No |
| 381 | CDC7 |  |  | 6788 | -0.045 | 0.1080 | No |
| 382 | NCAPD3 |  |  | 6797 | -0.045 | 0.1081 | No |
| 383 | CSE1L |  |  | 6810 | -0.045 | 0.1077 | No |
| 384 | C19orf48 |  |  | 6811 | -0.045 | 0.1087 | No |
| 385 | SGO1 |  |  | 6841 | -0.046 | 0.1066 | No |
| 386 | NUP85 |  |  | 6842 | -0.046 | 0.1075 | No |
| 387 | CENPF |  |  | 6902 | -0.047 | 0.1023 | No |
| 388 | MASTL |  |  | 6931 | -0.048 | 0.1003 | No |
| 389 | POLD3 |  |  | 7016 | -0.051 | 0.0924 | No |
| 390 | SNX5 |  |  | 7025 | -0.051 | 0.0927 | No |
| 391 | CENPJ |  |  | 7066 | -0.052 | 0.0895 | No |
| 392 | MCM8 |  |  | 7126 | -0.054 | 0.0844 | No |
| 393 | IKBIP |  |  | 7129 | -0.054 | 0.0853 | No |
| 394 | CDC6 |  |  | 7140 | -0.054 | 0.0854 | No |
| 395 | SMC3 |  |  | 7149 | -0.054 | 0.0857 | No |
| 396 | LMNB1 |  |  | 7166 | -0.055 | 0.0851 | No |
| 397 | FAM111B |  |  | 7169 | -0.055 | 0.0861 | No |
| 398 | EZH2 |  |  | 7260 | -0.057 | 0.0777 | No |
| 399 | OLFM1 |  |  | 7262 | -0.057 | 0.0788 | No |
| 400 | CCP110 |  |  | 7307 | -0.058 | 0.0753 | No |
| 401 | PRIM1 |  |  | 7308 | -0.058 | 0.0765 | No |
| 402 | MCM7 |  |  | 7397 | -0.061 | 0.0685 | No |
| 403 | MCM6 |  |  | 7414 | -0.061 | 0.0680 | No |
| 404 | KDELR2 |  |  | 7423 | -0.061 | 0.0685 | No |
| 405 | LIG1 |  |  | 7482 | -0.063 | 0.0636 | No |
| 406 | FANCD2 |  |  | 7483 | -0.063 | 0.0650 | No |
| 407 | KLHL23 |  |  | 7508 | -0.064 | 0.0638 | No |
| 408 | MNS1 |  |  | 7531 | -0.064 | 0.0628 | No |
| 409 | IDH2 |  |  | 7549 | -0.065 | 0.0623 | No |
| 410 | RRM1 |  |  | 7556 | -0.065 | 0.0631 | No |
| 411 | MCM4 |  |  | 7658 | -0.068 | 0.0538 | No |
| 412 | TOP2A |  |  | 7678 | -0.069 | 0.0532 | No |
| 413 | ARL6IP6 |  |  | 7747 | -0.072 | 0.0475 | No |
| 414 | CDC45 |  |  | 7751 | -0.072 | 0.0487 | No |
| 415 | PEG10 |  |  | 7803 | -0.073 | 0.0448 | No |
| 416 | C21orf58 |  |  | 7804 | -0.074 | 0.0463 | No |
| 417 | ASPM |  |  | 7821 | -0.074 | 0.0462 | No |
| 418 | BRIP1 |  |  | 7851 | -0.075 | 0.0447 | No |
| 419 | PARP1 |  |  | 7864 | -0.076 | 0.0450 | No |
| 420 | FUCA2 |  |  | 7914 | -0.078 | 0.0415 | No |
| 421 | HAT1 |  |  | 7958 | -0.080 | 0.0386 | No |
| 422 | BARD1 |  |  | 7980 | -0.080 | 0.0380 | No |
| 423 | DDB2 |  |  | 8121 | -0.086 | 0.0250 | No |
| 424 | CEP152 |  |  | 8127 | -0.086 | 0.0262 | No |
| 425 | RBL1 |  |  | 8134 | -0.087 | 0.0274 | No |
| 426 | TMEM97 |  |  | 8161 | -0.088 | 0.0265 | No |
| 427 | BRCA2 |  |  | 8192 | -0.089 | 0.0252 | No |
| 428 | RAD51AP1 |  |  | 8227 | -0.091 | 0.0235 | No |
| 429 | SLF1 |  |  | 8264 | -0.093 | 0.0216 | No |
| 430 | MIS18BP1 |  |  | 8274 | -0.093 | 0.0226 | No |
| 431 | CASP8AP2 |  |  | 8275 | -0.093 | 0.0246 | No |
| 432 | RFC4 |  |  | 8292 | -0.094 | 0.0249 | No |
| 433 | MCM10 |  |  | 8339 | -0.096 | 0.0220 | No |
| 434 | FAM111A |  |  | 8359 | -0.097 | 0.0220 | No |
| 435 | MSH2 |  |  | 8392 | -0.099 | 0.0207 | No |
| 436 | TMEM107 |  |  | 8394 | -0.099 | 0.0227 | No |
| 437 | PIGX |  |  | 8420 | -0.100 | 0.0221 | No |
| 438 | TMX1 |  |  | 8524 | -0.107 | 0.0134 | No |
| 439 | KIF15 |  |  | 8542 | -0.108 | 0.0139 | No |
| 440 | FAM161A |  |  | 8545 | -0.108 | 0.0160 | No |
| 441 | MMS22L |  |  | 8547 | -0.108 | 0.0181 | No |
| 442 | ESCO2 |  |  | 8566 | -0.109 | 0.0185 | No |
| 443 | RDX |  |  | 8619 | -0.112 | 0.0154 | No |
| 444 | CEP135 |  |  | 8623 | -0.113 | 0.0174 | No |
| 445 | GGH |  |  | 8643 | -0.114 | 0.0178 | No |
| 446 | SMC1A |  |  | 8681 | -0.116 | 0.0163 | No |
| 447 | DEGS1 |  |  | 8698 | -0.117 | 0.0171 | No |
| 448 | KDELC2 |  |  | 8703 | -0.118 | 0.0191 | No |
| 449 | SMC2 |  |  | 8721 | -0.119 | 0.0198 | No |
| 450 | NFATC2IP |  |  | 8739 | -0.121 | 0.0206 | No |
| 451 | CBX5 |  |  | 8780 | -0.124 | 0.0189 | No |
| 452 | HIST1H4C |  |  | 8820 | -0.126 | 0.0174 | No |
| 453 | CDCA7 |  |  | 8824 | -0.126 | 0.0198 | No |
| 454 | SPATA33 |  |  | 8885 | -0.133 | 0.0162 | No |
| 455 | ORC6 |  |  | 8888 | -0.133 | 0.0188 | No |
| 456 | FANCI |  |  | 8917 | -0.136 | 0.0187 | No |
| 457 | GINS4 |  |  | 8919 | -0.136 | 0.0214 | No |
| 458 | FANCA |  |  | 8933 | -0.137 | 0.0229 | No |
| 459 | ATAD2 |  |  | 8948 | -0.138 | 0.0244 | No |
| 460 | ATAD5 |  |  | 9000 | -0.142 | 0.0219 | No |
| 461 | TCEAL9 |  |  | 9041 | -0.146 | 0.0208 | No |
| 462 | FADS1 |  |  | 9044 | -0.147 | 0.0236 | No |
| 463 | LRRCC1 |  |  | 9082 | -0.150 | 0.0229 | No |
| 464 | RBBP4 |  |  | 9262 | -0.174 | 0.0075 | No |
| 465 | ABHD3 |  |  | 9348 | -0.189 | 0.0025 | No |
| 466 | METRN |  |  | 9381 | -0.196 | 0.0032 | No |
| 467 | HELLS |  |  | 9406 | -0.201 | 0.0049 | No |
| 468 | CALU |  |  | 9418 | -0.205 | 0.0080 | No |
| 469 | PRKDC |  |  | 9539 | -0.236 | 0.0002 | No |
| 470 | BRCA1 |  |  | 9600 | -0.258 | -0.0007 | No |
| 471 | XRCC2 |  |  | 9653 | -0.284 | -0.0003 | No |
| 472 | EMP2 |  |  | 9696 | -0.310 | 0.0018 | No |
| 473 | HACD3 |  |  | 9697 | -0.311 | 0.0084 | No |
| 474 | PGRMC1 |  |  | 9826 | -0.516 | 0.0056 | No |
Table: GSEA details [plain text format]

  

Fig 2: DOROSZKO\_U3180MG\_CLUSTER\_3: Random ES distribution      
 Gene set null distribution of ES for **DOROSZKO\_U3180MG\_CLUSTER\_3**
